# From Blue Light to Clock Genes in Zebrafish ZEM-2S Cells

**DOI:** 10.1371/journal.pone.0106252

**Published:** 2014-09-03

**Authors:** Bruno C. R. Ramos, Maria Nathália C. M. Moraes, Maristela O. Poletini, Leonardo H. R. G. Lima, Ana Maria L. Castrucci

**Affiliations:** 1 Department of Physiology, Institute of Biosciences, University of São Paulo, São Paulo, Brazil; 2 Department of Physiology, Institute of Biological Sciences, Federal University of Minas Gerais, Belo Horizonte, Brazil; University of Ferrara, Italy

## Abstract

Melanopsin has been implicated in the mammalian photoentrainment by blue light. This photopigment, which maximally absorbs light at wavelengths between 470 and 480 nm depending on the species, is found in the retina of all classes of vertebrates so far studied. In mammals, melanopsin activation triggers a signaling pathway which resets the circadian clock in the suprachiasmatic nucleus (SCN). Unlike mammals, *Drosophila melanogaster* and *Danio rerio* do not rely only on their eyes to perceive light, in fact their whole body may be capable of detecting light and entraining their circadian clock. Melanopsin, teleost multiple tissue (tmt) opsin and others such as neuropsin and va-opsin, are found in the peripheral tissues of *Danio rerio*, however, there are limited data concerning the photopigment/s or the signaling pathway/s directly involved in light detection. Here, we demonstrate that melanopsin is a strong candidate to mediate synchronization of zebrafish cells. The deduced amino acid sequence of melanopsin, although being a vertebrate opsin, is more similar to invertebrate than vertebrate photopigments, and melanopsin photostimulation triggers the phosphoinositide pathway through activation of a G_q/11_-type G protein. We stimulated cultured ZEM-2S cells with blue light at wavelengths consistent with melanopsin maximal absorption, and evaluated the time course expression of *per1b*, *cry1b*, *per2 and cry1a*. Using quantitative PCR, we showed that blue light is capable of slightly modulating *per1b* and *cry1b* genes, and drastically increasing *per2 and cry1a* expression. Pharmacological assays indicated that *per2* and *cry1a* responses to blue light are evoked through the activation of the phosphoinositide pathway, which crosstalks with nitric oxide (NO) and mitogen activated protein MAP kinase (MAPK) to activate the clock genes. Our results suggest that melanopsin may be important in mediating the photoresponse in *Danio rerio* ZEM-2S cells, and provide new insights about the modulation of clock genes in peripheral clocks.

## Introduction

Melanopsin was discovered in 1998 by Provencio and coworkers [1] in *Xenopus leavis* melanophores. This opsin arose as a strong candidate to mediate the synchronization process of the biological clock to light because it was found not only in the skin but also in the retina of this animal. In fact, further studies confirmed the presence of this opsin in the retina of all vertebrates studied to date [2]–[8]. So far, its major role in the entrainment of the biological clock has only been demonstrated in mammals [9]–[14]. Although an important vertebrate photopigment, melanopsin shares sequence identity with invertebrate opsins present in rhabdomeric photoreceptors [1],[5].

Rhabdomeric photoreceptor signaling was mainly studied in *Drosophila melanogaster* [15] and it involves the activation of G_q/11_ protein, phospholipase C (PLC) and subsequent opening of transient receptor potential channels (TRPCs), resulting in membrane depolarization. Similar signaling pathway has been proposed for melanopsin. Heterologous expression of melanopsin in *Xenopus* sp. oocytes, *Cercopithecus aethiops* fibroblast-like kidney (COS), *Mus musculus* neuroblastoma (Neuro-2A) and *Homo sapiens* embryonic kidney (HEK-293) cells rendered these cells photosensitive [7], [16]–[19]. Antagonists and antibodies against G_q/11_ abolished or greatly attenuated the melanopsin response to light [17], [19]. Furthermore different groups have demonstrated that melanopsin activates PLC with a subsequent production of inositol-3-phosphate (IP_3_), a raise in intracellular calcium, and protein kinase C (PKC) activation in both native [20]–[23] and heterologous [19], [24] systems.

Although the role of melanopsin in the setting of the central mammalian clock and its signaling pathway have been partially elucidated, many questions remain unanswered regarding the transduction mechanisms of melanopsin and other pigments, such as Rgr and Tmt, in peripheral clocks. Tmt has been a major candidate to modulate peripheral clocks [25], [26]. This study chose to investigate the involvement of the melanopsin signaling pathway in the regulation of clock genes by blue light in ZEM-2S cells.

The molecular mechanism of the circadian clock is based on feedback loops of cycling gene products, which control their own synthesis through gene and protein negative and positive regulation [27]–[29]. In mammals, the heterodimer composed of BMAL1 (brain and muscle Arnt-like protein 1) and CLOCK/NPAS2 (neuronal PAS domain protein 2) is a transcriptional activator that regulates transcription of *Per* (period) and *Cry* (cryptochrome) genes which encode the repressors of BMAL1 heterodimer activity, thus closing a negative feedback loop that generates rhythms of approximately 24 h [30], [31]. Clock genes are not only expressed in the central circadian pacemaker of mammals, but also in the majority of cell types from many different organisms [32]. In some organisms such as the fruit fly (*Drosophila* sp.) and the teleost *Danio rerio*, the clocks in the tissues can be directly synchronized by light [33]–[36] which raises a new issue about the hierarchy between the central oscillator and the peripheral clocks. Indeed, even clocks in cultured cells can be synchronized by light [36]–[38] and other factors, such as temperature [39] and serum shock [40], making them a great model to study peripheral clocks. Although still unproved, melanopsin may be the photopigment in these cells, as it has been found not only in the retina of all classes of vertebrates, but in peripheral tissues of *Gadus morhua* (cod) [41], *Xenopus laevis* (clawed frog) [20] and *Gallus gallus* (chicken) [42], and its presence may reflect a major role in photosynchronization of the peripheral clocks in these animals.

The popular zebrafish, *Danio rerio*, has been used for decades as a model to study vertebrate development; more recently, however, its use has rapidly expanded, and it has become a model of visual systems [43], pain mechanisms [44], diseases and the discovery of new drugs [45] as well. The zebrafish has also been useful to study the genetics of the circadian clock [46]–[48], particularly because clock in embryo-derived cell lines can be synchronized by light [36]–[38]. Although this species possesses six *cry* and four *per* genes, its core mechanism closely resembles the mammalian molecular clock [49]. It is important to mention that *Danio rerio* expresses five melanopsins (*opn4m-1*, *opn4m-2*, *opn4m-3*, *opn4x-1 and opn4x-2*) [7] and other opsins such as tmt opsin and va opsin (vertebrate ancient opsin), all of them being considered candidates to mediate the resetting of clock genes by light. Besides opsins, there are two other possible candidates by which light could reach clock genes and fulfill the role of the “circadian light-sensor”: (I) Photosensitive *cry* (cryptochrome) proteins and (II) flavin-containing oxidases. Despite the number of candidates, there has been no consensus about the nature of the photopigment responsible for resetting the clock genes in zebrafish.

Here we provide evidence that an opsin, in this case melanopsin, may mediate the photoentrainment of clock genes in zebrafish cells. Our results showing the influence of blue light on clock gene expression and the participation of the phosphoinositide pathway in this response are consistent with what is currently known about melanopsin. Furthermore we also show that clock gene induction depends on NO and mitogen-activated protein kinase (MAPK).

## Materials and Methods

### ZEM-2S cell culture

Fibroblast-like embryonic cells of *Danio rerio* (ZEM-2S) (kindly donated by Prof. Mark Rollag, Uniformed Services University of the Health Sciences, USA, originally purchased from ATCC, CRL-2147, Manassas, VA, USA) were maintained at 28°C in 50% Leibovitz L-15, 35% Dulbecco's Modified Eagle medium (D-MEM), 15% Ham's F12, and 15 mM 4-(2-Hydroxyethyl)-1-piperazineethanesulfonic acid (HEPES) (Life Technologies, Carlsbad, CA, USA), complemented with 10% fetal calf serum (Emcare, Campinas, SP, Brasil) and 1% antibiotic/antimycotic (10,000 U/mL penicillin; 10,000 µg/mL streptomycin; 25 µg/mL amphotericin B). Culture medium was changed twice a week and cells were harvested with Tyrode/ethylenediaminetetraacetic acid (EDTA) solution (NaCl 8.0 g/L; KCl 0.2 g/L; NaHCO_3_ 1.0 g/L; NaH_2_PO_4_ 0.05 g/L; MgCl_2_ 1.0 g/L; EDTA 1.86 g/L) and subcultured (1∶3 dilution) when 80% confluent.

Before the experiments, the serum concentration was reduced to 2% and 200 nM all-trans retinaldehyde (Sigma, St. Louis, MO, USA) was added. This experimental set-up has been established taking in account that (1) all the reactions to regenerate the chromophore have to happen within the same cell type; three among the five zebrafish melanopsins are bistable [7], that is the regeneration of 11-cis from all-trans chromophore occurs *in situ*, in response to a different wavelength, a typical mechanism of rhabdomeric opsins; (2) all 5 melanopsins of *D. rerio* transfected into Neuro-2A cells exhibit a better response to light if the assay is supplemented with the aldehyde [7]; (3) serum concentration in ZEM-2S cells was reduced to 2% during the assays. Cells were handled in the dark under a red safelight (7 W Konex bulb and Safe-Light filter GBX-2, Kodak, Rochester, NY, USA).

### Blue light stimulation

#### Protocol 1

ZEM-2S cells were seeded (2×10∧6 cells/25 cm^2^ flask), placed in constant dark (DD) for 6 d, and stimulated with blue light (450–475 nm, λ peak = 463 nm, 87.85 to 95.17 µwatts/cm^2^) for 10 min at the beginning of day 7. The cells were then kept in DD and total RNA was extracted 1, 2, 6 and 12 h after the light pulse. A control group (without blue light pulse) was kept in DD throughout the experiment and RNA was extracted at the same time points. Although the cells were kept previously and throughout the experiments in DD, caution was taken to obtain the samples at the same time of the day in this and the following protocols.

#### Protocol 2

To investigate the signaling pathway evoking the increase in clock gene expression, the cells were separated in four groups: (I) DD; (II) DD in the presence of specific inhibitors; (III) submitted to a light pulse as in protocol 1; and (IV) submitted to a light pulse in the presence of an inhibitor. The inhibitors were added to the cells 30 min before light stimulation and remained in the flasks throughout the experiment. The following inhibitors were used: 1-[6-[[(17β)-3-methoxyestra-1,3,5(10)-trien-17-yl]amino]hexyl]-1*H*-pyrrole-2,5-dione (U-73122, phospholipase C, PLC, inhibitor); 1,2-bis(o-aminophenoxy)ethane-N,N,N′,N′-tetraacetic acid (BAPTA-AM, calcium chelator); 2-{1-[3-(amidinothio)propyl]-1H-indol-3-yl}-3-(1-methylindol-3-yl)maleimide methanesulfonate salt (RO 31-8220, protein kinase C, PKC, inhibitor); *N*-[2-[[[3-(4-chlorophenyl)-2-propenyl]methylamino]methyl]phenyl]-*N*-(2-hydroxyethyl)-4-methoxybenzenesulphonamide (KN-93, calcium/calmodulin kinase II, CAMK II, inhibitor); L-NG-nitroarginine methyl ester (L-NAME, NO synthase, NOS, inhibitor); 2-(2-amino-3-methoxyphenyl)-4H-1-benzopyran-4-one (PD-98059, mitogen-activated kinase kinase, MEK, inhibitor), and 9-(tetrahydro-2-furanyl)-9H-purin-6-amine (SQ-22536, adenylyl cyclase inhibitor), all from Enzo Life Sciences, Plymouth Meeting, PA, USA. Stock solutions were made in DMSO (maximal concentration in the culture medium was 0.1%), except for L-NAME and SQ-22536, which were dissolved in sterile water. The vehicles were previously tested and had no effect on the expression of the studied genes.

#### Protocol 3

We have previously shown in *Xenopus laevis* melanophores that light induces melanin dispersion through a phosphoinositide pathway. Interestingly, cGMP was also produced in response to light, but its permeable analogue did not elicit melanin translocation nor did the blockade of PKG affect the photoresponse [20]. Aiming to investigate the role of cGMP and whether NO is an intracellular messenger induced by light, we kept cells in DD as in protocol 1, and the guanylyl cyclase stimulator, 5-[1-(phenylmethyl)-1H-indazol-3-yl]-2-furanmethanol (YC-1, Enzo Life Sciences, Plymouth Meeting, PA, USA, dissolved in sterile water), at 40 µM, was added to the cells for 30 min at the beginning of day 7, and remained in the preparation throughout the experiment. Total RNA was extracted 2 h after the end of the treatment. Appropriate controls were as follows: negative control, no light/no YC-1; positive control, blue light/no YC-1.

#### Protocol 4

ZEM-2S cells were seeded (8×10∧5 cells/well in a 96 well plate) and placed in constant dark (DD) for 3 d. Cells were treated with 100 µM IBMX (3-isobutyl-1-methylxanthine, Enzo Life Science, Plymouth Meeting, PA, USA) 30 min prior to stimulation with blue light (450–475 nm, 87.85 to 95.17 µ watts/cm^2^) for 1, 5 and 10 min. As a positive control, 10 µM forskolin was applied for 15 min in dark-kept cells. After stimulation, cells were lysed, and the samples assayed according to the manufacturer's protocol (*Direct 3′-5′-cyclic adenosine monophosphate enzyme-linked immunosorbent assay (ELISA) kit*, Enzo Life Sciences, Plymouth Meeting, PA, USA). The nucleotide concentrations were compared by one-way ANOVA, followed by Tukey, and the difference was considered significant when p<0.05.

### Total RNA extraction and RT-PCR

The medium was discarded, 1 mL of *Tri-Reagent-LS* (Life Technologies, Carlsbad, CA, USA) was added directly to the cells, and RNA was obtained according to the manufacturer's instructions. Following ethanol rinses, the RNA pellet was dried, resuspended in 20 µL of diethylpyrocarbonate H_2_O (Life Technologies, Carlsbad, CA, USA) and treated with DNase I according to the manufacturer's instructions (turbo-*DNA-free*, Life Technologies, Carlsbad, CA, USA). Total RNA concentration (absorbance at 260 nm) and the RNA quality (ratio A260/A280) were determined by using a Nanodrop spectrophotometer, Thermo Scientific, Wilmington, DE, USA), and RT-PCR was performed with 1 µg of total RNA, utilizing 1 µL of random primers (300 µg/µL, 6-mer) and Superscript III (200 U/µL, Life Technologies, Carlsbad, CA, USA), using the following protocol: 65°C for 5 min, kept on ice for 1 min; then, after the addition of the reverse transcriptase enzyme, 5 min at 25°C, 50 min at 50°C, and 15 min at 70°C.

### Quantitative PCR

It has been reported that four (Cry1a, Cry1b, Cry2a, and Cry2b) among the 6 Cry proteins of zebrafish are able to inhibit mammalian CLOCK∶BMAL1 activity. In zebrafish eye, brain and body the mRNA rhythms of *cry1a* and *cry1b* peak during the daytime, whereas *cry2a* and *cry2b* peak in the evening. As to *per* genes, in zebrafish Z3 cells, *per1* and *per3* rhythms persist in constant conditions, whereas *per2* mRNA is stimulated by light, but it is not rhythmic in constant conditions [46]. So, we chose to determine the photoresponse of the two *cry* genes, *cry1a* and *cry1b*, that peak in the daylight, and of a rhythmic and a light-stimulated *per* gene, respectively *per1* and *per2*.

The solutions for quantitative PCR contained the primers and fluorescent probes, as shown in Table 1, and Supermix 1× (Life Technologies, Carlsbad, CA, USA) supplemented to final concentrations of 400 µM each dNTP, 6 mM MgCl_2_ and 0.1 U/µL Platinum *Taq* DNA polymerase (Life Technologies, USA). This solution was aliquoted over three wells and 1 ul of cDNA of each sample was added to each well. Each experimental cDNA was run in triplicates in 96 well plates.

**Table 1:** *Danio rerio* primers and probes for quantitative PCR.

	Sequences	Final concentration
18S rRNA X03205.1	For: 5′–CGGCTACCACATCCAAGGAA–3′	50 nM
	Rev: 5′–GCTGGAATTACCGCGGCT–3′	50 nM
	Pr:5′–/5TexRd/TGCTGGCACCAGACTTGCCCTC/3BHQ_2/–3′	50 nM
*per1* AY597250 or M_212439.2	For: 5′–AGCTCAAACTCTCACAGCCCTT–3′	300 nM
	Rev: 5′– TCAGAGCTGGCACTCAACAGA –3′	300 nM
	Pr:5′–/5Cy5/TCCACCCAGCAGTTCTCTGGCATACA/3BHQ_2/–3′	200 nM
*cry1b* NM_131790.4	For: 5′–CGTCTCTGGAGGAGCTCGG–3′	300 nM
	Rev: 5′– TCTCCCCCGGGCCAC–3′	300 nM
	Pr:5′–/5HEX/TTTGAAACAGAGGGACTGTCCACTGCTG/3BHQ_1/–3′	200 nM
*per2* AY597250 or NM_212439.2	For: 5′– GTGGAGAAAGCGGGCAGC–3′	300 nM
	Rev:5′–GCTCTTGTTGCTGCTTTCAGTTCT–3′	300 nM
	Pr:5′/6FAM/ATGGGTTCAGGATCAAACCGCTGT/3BHQ_1/3′	200 nM
*cry1a* NM_131790.4	For:5′–CTACAGGAAGGTCAAAAAGAACAGC–3′	300 nM
	Rev: 5′–CTCCTCGAACACCTTCATGCC–3′	300 nM
	Pr:5′–/5HEX/AAAGCGTGGGTTGTTTGTAGCAGC/3BHQ_1/–3′	200 nM

For = forward primer; Rev = reverse primer; Pr = fluorescent probe; TexRd = Texas Red; Cy5 = Cyanine 5; Hex = 6-carboxy-2, 4,4, 5, 7,7 -hexachlorofluorescein succinimidyl ester; FAM = Carboxyfluorescein; 3BHQ_1 = Black hole quencher 1 (IDT); 3BHQ_2 = Black hole quencher 2 (IDT).

The oligonucleotides (Table 1) were designed using the Primer Express program (Life Technologies, Carlsbad, CA, USA), based on sequences obtained from GenBank (http://www.ncbi.nlm.nih.gov/GenBank), and synthesized by IDT (Coralville, IA, USA). Primer efficiencies were determined to be higher than 80%. 18S rRNA was utilized to normalize the values of the studied genes, as widely used [50]–[53], in order to correct for pipetting errors. All assays were performed using an iQ5 (BioRad, Hercules, CA, USA) thermocycler, with the following protocol: 7 min at 95°C followed by 50 cycles of 10 sec at 95°C and 1 min at 60°C.

Data analysis was based on the ΔΔC_T_ method [54], and compared the number of cycles between control and experimental wells, by passing a threshold line through the geometric portions of the amplification curves. ΔC_T_, the difference between these values for the gene of interest and 18S rRNA, was then calculated. Next, the mean value of ΔC_T_ for control wells was subtracted from the experimental values, originating the ΔΔC_T_. This value was then used as the negative exponential of base 2, averaged from at least four flasks of cells, from two independent experiments. The log data were analyzed by one-way ANOVA, followed by Tukey, and the difference was considered significant when p<0.05.

## Results

**Figure 1:**
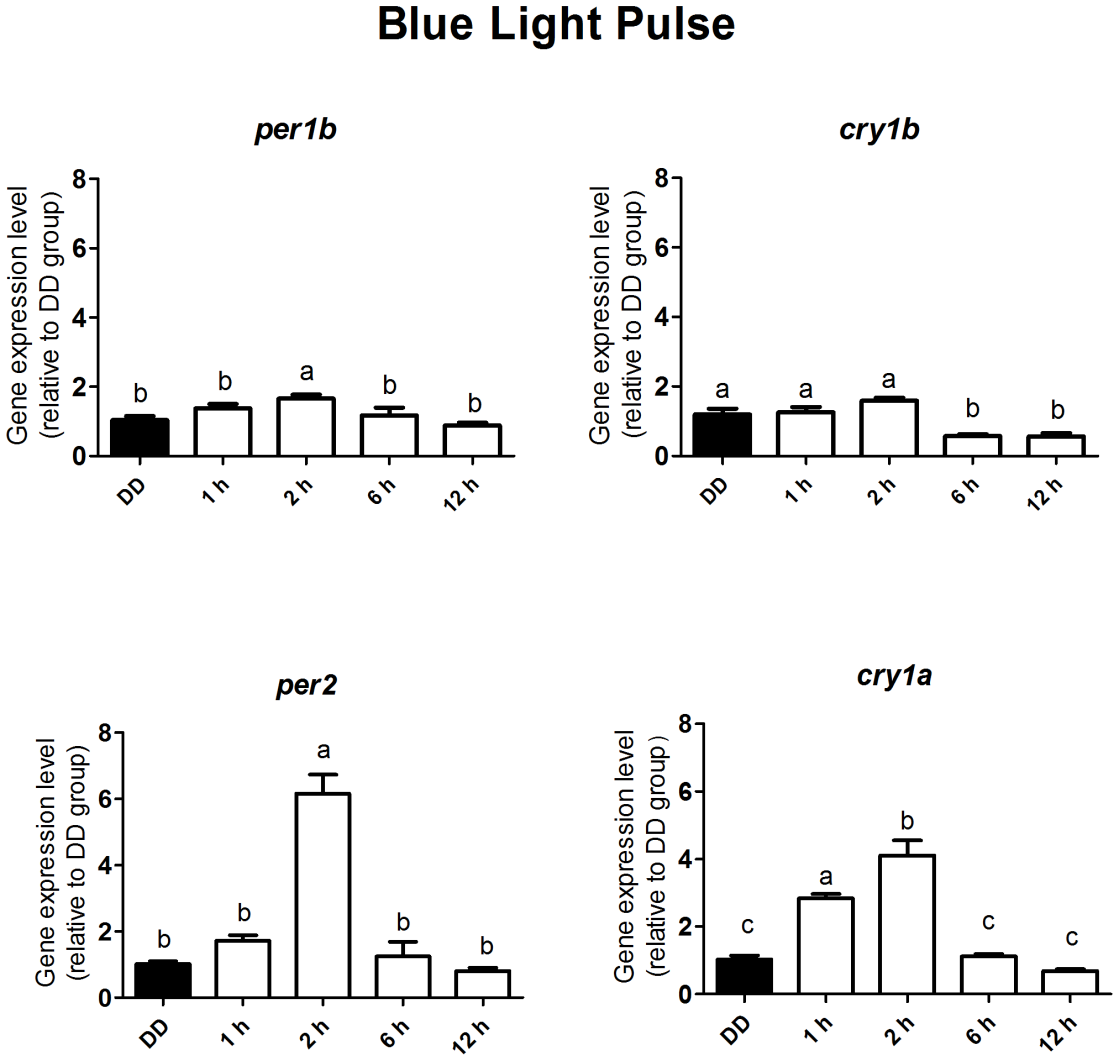
Quantitative PCR of *per1b*, *cry1b*, *per2* and *cry1a* in a *Danio rerio* embryonic cell line ZEM-2S. The cells (2×10∧6) were stimulated with blue light (450–475 nm, 87.85 to 95.17 µwatts/cm^2^) for 10 min, and total RNA was extracted 1, 2, 6 and 12 h after the stimulus. ‘a’ is significantly different from ‘b’ and ‘b’ is significantly different from ‘c’ (p<0.05). In this and in figures 2 to 9, values are the mean ± Standard Error of Mean (n = 4–9).

Previous work from our group demonstrated that ZEM-2S embryonic cells are photosensitive and display differential growth under different light/dark regimens [38]. In that study two important discoveries led us to the present investigation. First, the presence of two melanopsin genes, initially mislabeled *opn4m* and *opn4x* (now known as *opn4m-1* and *opn4m-2*, respectively) in ZEM-2S cells; second, *per1* and *cry1b* expression did not vary under constant dark but displayed robust rhythms in conditions of light-dark cycles. According to the literature [7], zebrafish has five melanopsin genes, so we decided to investigate whether ZEM-2S cells expressed all five melanopsins, and we found the following expressions (average C_T_s of 6 time points along 24 h, determined in 3–6 flasks of cells): *opn4m-2* = 30.9>*opn4m-1* = 31.7>*opn4m-3* = 33.3>*opn4x-2* = 35.4>*opn4x-1* = 35.8. We then asked whether blue light would effectively mimic what white light does, what would suggest melanopsin as one of the putative candidates for clock gene regulation in ZEM-2S cells. Our first step was to evaluate the gene expression of *per1b*, *per2*, *cry1a* and *cry1b* for up to 12 h after a 10 min blue light (450–475 nm) pulse, a range of wavelengths that spectrally overlap with the absorption spectrum of melanopsin. It is worth to mention that the five *Danio rerio* melanopsins may exhibit peak sensitivities varying between 470 and 484 nm [7]. According to the literature, *per1b* and *cry1b* are synchronized by light [46], [49] and *per2* and *cry1a* are inducible by light [55], [56]. Our results show that *per1b* and c*ry1b* can be altered by blue light pulse (Fig. 1): *per1b* expression slightly increased after two hours (p<0.0083) and c*ry1b* expression slightly decreased after six hours (p<0.0001). Although responsive to the light stimulus, the responses of these genes were much smaller than what was seen for *per2* and *cry1a* (Fig. 1). The expression of *per2* increased about 6-fold two hours after the light pulse (p<0.0001), and *cry1a* mRNA increased about 4-fold at the same time point (p<0.0001), both returning to basal level after six hours.

**Figure 2:**
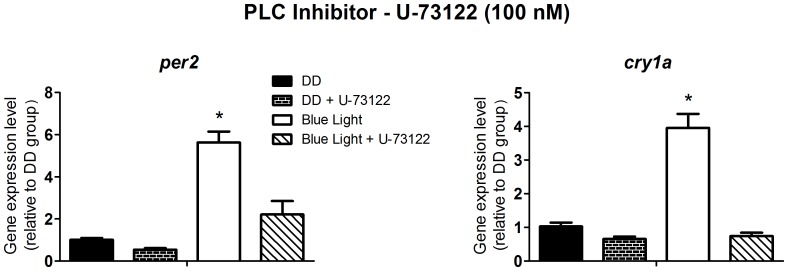
Quantitative PCR of *per2* and *cry1a* in a *Danio rerio* embryonic cell line ZEM-2S. The cells (2×10∧6) were stimulated with blue light (450–475 nm, 87.85 to 95.17 µwatts/cm^2^) for 10 min, in the presence or absence of the PLC inhibitor, U-73122, at 100 nM, and total RNA was extracted 2 h after the stimulus. In this and the following figures, the asterisk means statistically significant differences from all other groups (p<0.05).

**Figure 3:**
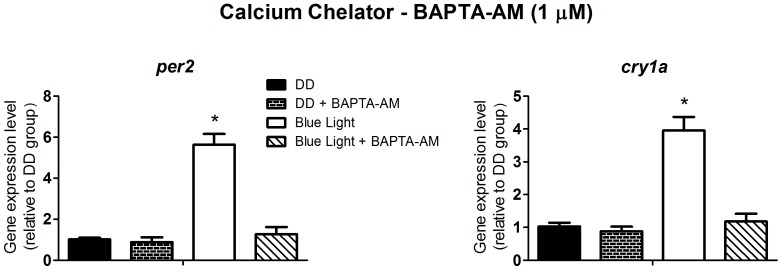
Quantitative PCR of *per2* and *cry1a* in a *Danio rerio* embryonic cell line ZEM-2S. The cells (2×10∧6) were stimulated with blue light (450–475 nm, 87.85 to 95.17 µwatts/cm^2^) for 10 min, in the presence or absence of the calcium chelator, BAPTA-AM, at 1 µM, and total RNA was extracted 2 h after the stimulus.

**Figure 4:**
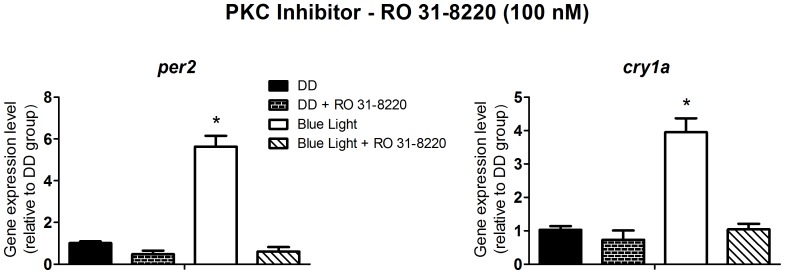
Quantitative PCR of *per2* and *cry1a* in a *Danio rerio* embryonic cell line ZEM-2S. The cells (2×10∧6) were stimulated with blue light (450–475 nm, 87.85 to 95.17 µwatts/cm^2^) for 10 min, in the presence or absence of the PKC inhibitor, Ro 31-8220, at 100 nM and total RNA was extracted 2 h after the stimulus.

To investigate light signaling we decided, therefore, to use *per2* and *cry1a* expression as the output of the experimental system, at the time point of their maximal response, i.e. two hours after the blue light pulse. Albeit a blue light pulse could modulate these clock genes, this is not an irrefutable proof that melanopsin, in fact, mediates this response, since other opsins such as va-opsin, pinopsin, encephalopsin/tmts have maximal absorbance spectra within the range we used in this study. So, in addition, we investigated the signaling pathway evoked by blue light using pharmacological approaches and evaluating the impact of blocking some key steps of the putative melanopsin phototransduction cascade on the expression of multiple clock genes. According to what is known from our studies in *Xenopus laevis* melanophores [20], where melanopsin mediates the melanosome photodispersion response through the phosphoinositide cascade, our hypothesis was that light-stimulated melanopsin in ZEM-2S cells would affect clock genes through the same pathway. Although both cell lines are embryo-derived, one has to bear in mind that they are in different developmental stages and come from distinct vertebrate classes. The phospholipase C inhibitor, U-73122, at 100 nM (Fig. 2); 1 µM M BAPTA-AM, a potent calcium chelator (Fig. 3); and 100 nM Ro 31-8220, a protein kinase C inhibitor (Fig. 4) abolished the responses evoked by blue light on *per2* and *cry1a* expression (p<0.0001). Despite demonstrating the participation of the phosphoinositide cascade in clock gene modulation, one further question remained: Which mechanism conveys this signaling pathway to gene expression?

In the mammalian SCN, few candidates fulfill this role. The involvement of NO/cGMP, MAPK and cAMP/protein kinase A (PKA) pathways has been extensively described in the literature to control and modulate the circadian response of the SCN [57]. So, our next step was to investigate the possibility of a crosstalk between the phosphoinositide cascade and these other signaling pathways.

**Figure 5:**
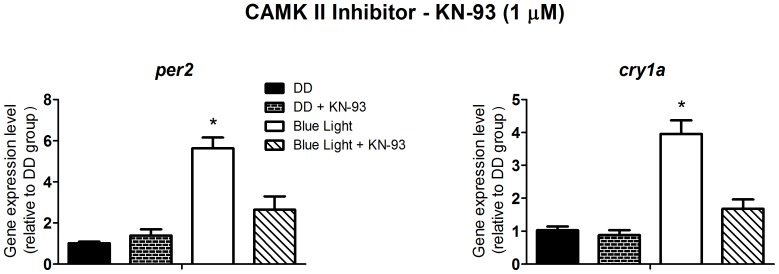
Quantitative PCR of *per2* and *cry1a* in a *Danio rerio* embryonic cell line ZEM-2S. The cells (2×10∧6) were stimulated with blue light (450–475 nm, 87.85 to 95.17 µwatts/cm^2^) for 10 min, in the presence or absence of the CAMK II inhibitor, KN-93, at 1 µM, and total RNA was extracted 2 h after the stimulus.

**Figure 6:**
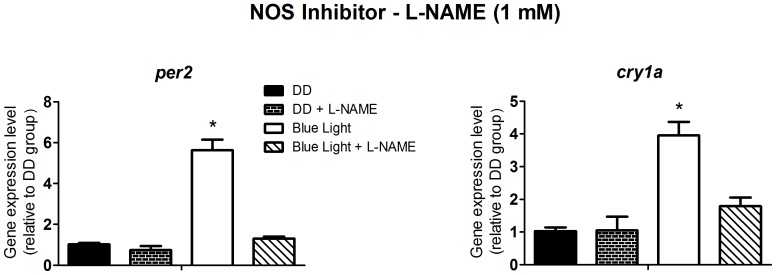
Quantitative PCR of *per2* and *cry1a* in a *Danio rerio* embryonic cell line ZEM-2S. The cells (2×10∧6) were stimulated with blue light (450–475 nm, 87.85 to 95.17 µwatts/cm^2^) for 10 min, in the presence or absence of the NOS inhibitor, L-NAME, at 1 mM and total RNA was extracted 2 h after the stimulus.

**Figure 7:**
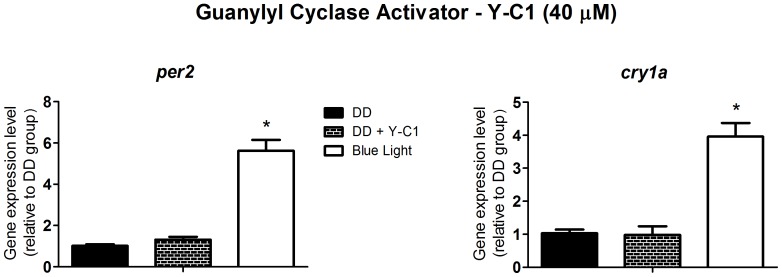
Quantitative PCR of *per2* and *cry1a* in a *Danio rerio* embryonic cell line ZEM-2S. The cells (2×10∧6) were stimulated with the guanylyl cyclase activator, Y-C1, at 40 µM in DD, and total RNA was extracted after 2 h.

From the previously mentioned study in *Xenopus laevis* [20], emerged a possible candidate to crosstalk with the PLC pathway. Isoldi and coworkers showed a 3-fold cGMP increase in *Xenopus* melanophores upon white light stimulation. Interestingly, the cyclic nucleotide does not take part in the light-induced pigment dispersion, as the blockade of cGMP production did not affect this response. Thus, we decided to evaluate whether the NO/cGMP pathway was involved with the induction of *per2* and *cry1a*. It is well known that CAMK II activates NO synthase resulting in NO production [58]. Therefore we decided to use the CAMK II inhibitor, KN-93, to investigate the participation of NO in this response. KN-93 at 1 µM (Fig. 5) inhibited the light-induced increase of *per2* and *cry1a* expression (p<0.0001), and 1 mM L-NAME, a non-specific inhibitor of NOS (Fig. 6), prevented the increase of *per2* and *cry1a* expression (p<0.0001). Curiously, despite the apparent role of NOS in the light response, the guanylyl cyclase activator, Y-C1, at 40 µM, had no effect on *per2* and *cry1a* expression, in cells maintained in DD (Fig. 7), suggesting that NO is probably acting through a cGMP-independent pathway.

**Figure 8:**
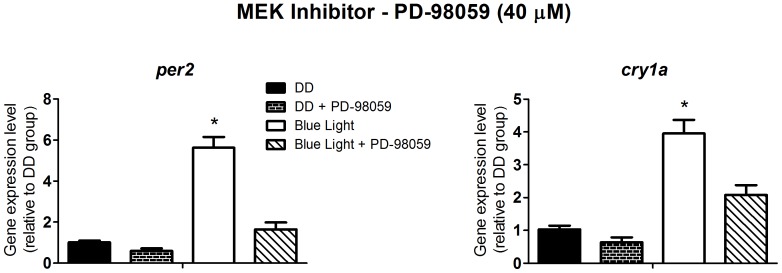
Quantitative PCR of *per2* and *cry1a* in a *Danio rerio* embryonic cell line ZEM-2S. The cells (2×10∧6) were stimulated with blue light (450–475 nm, 87.85 to 95.17 µwatts/cm^2^) for 10 min, in the presence or absence of the MEK inhibitor, PD-98059 at 40 µM, and total RNA was extracted 2 h after the stimulus.

The MAPK/ERK signaling pathway has already been implicated in the increased expression of *per2* in response to white light in *Danio rerio* [59]; our results corroborate these data for blue light, since the inhibitor of MEK, PD-98059 at 40 µM, blocked the rise in expression of *per2* and *cry1a* (p<0.0001) in response to the blue light pulse (Fig. 8).

**Figure 9:**
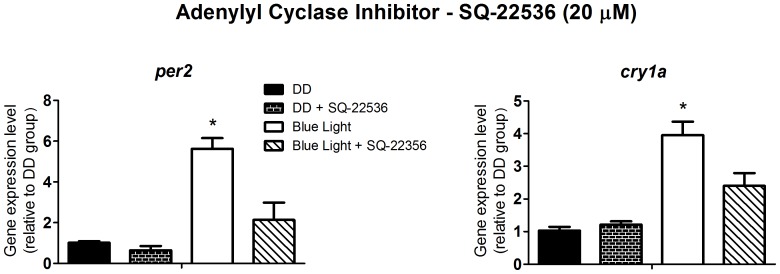
Quantitative PCR of *per2* and *cry1a* in a *Danio rerio* embryonic cell line ZEM-2S. The cells (2×10∧6) were stimulated with blue light (450–475 nm, 87.85 to 95.17 µwatts/cm^2^) for 10 min, in the presence or absence of the adenylyl cyclase inhibitor, SQ-22536, at 20 µM, and total RNA was extracted 2 h after the stimulus.

**Figure 10:**
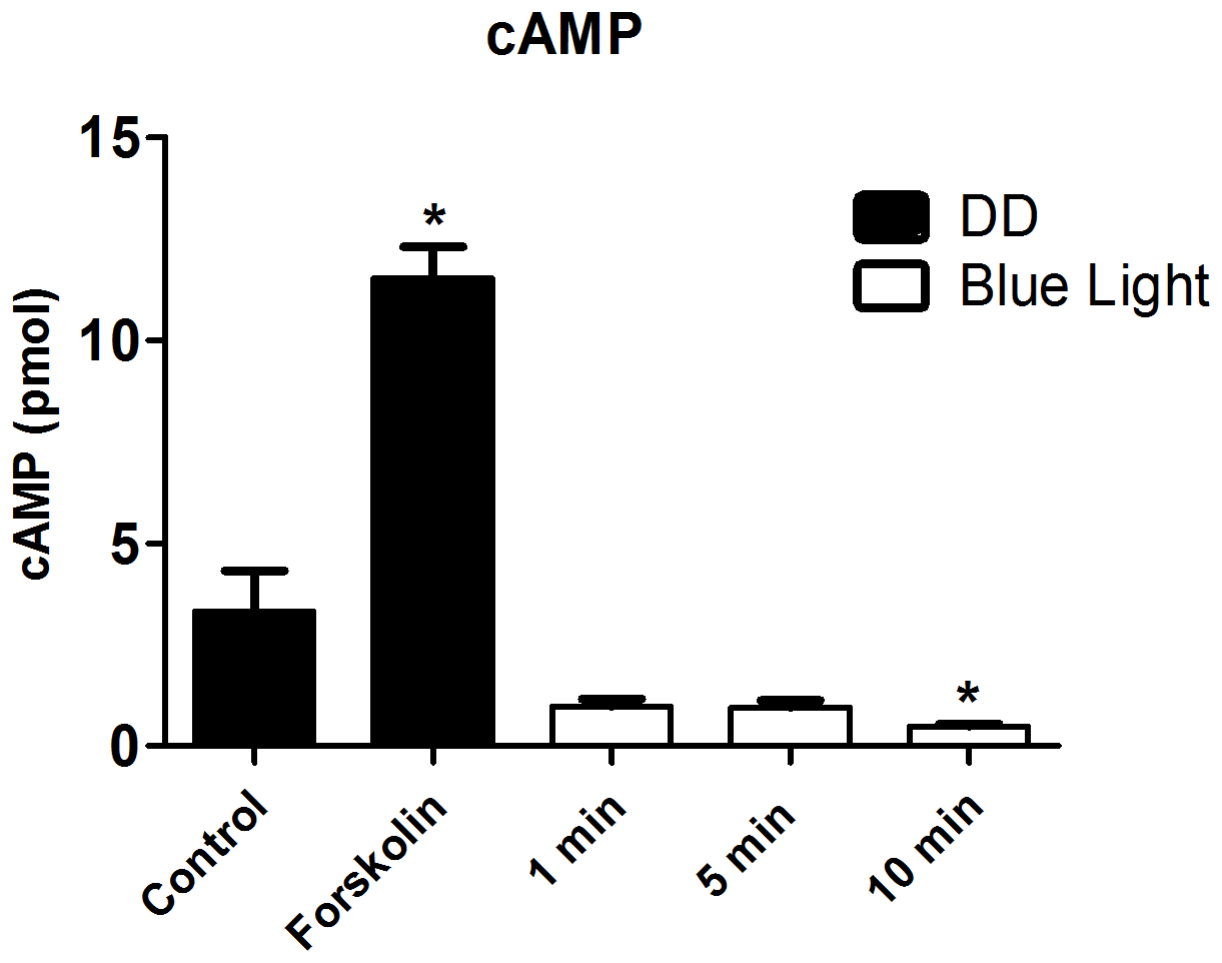
Quantification of cAMP in a *Danio rerio* embryonic cell line ZEM-2S. The cells (8×10∧4) were stimulated with blue light (450–475 nm, 87.85 to 95.17 µwatts/cm^2^) for 1, 5 or 10 min, and cAMP was measured immediately after each light pulse. Forskolin at 10 µM was used as a positive control in DD. Values are the mean ± SEM (n = 3).

In addition, we evaluated the cAMP/PKA pathway. The inhibitor of adenylyl cyclase, SQ-22536 at 20 µM, decreased *per2* and *cry1a* expression (p<0.0001, Fig. 9), and the quantification of cAMP, 10 min after the blue light pulse, shows that light, rather than increasing the nucleotide concentration, actually decreased cAMP concentration (Fig. 10). This effect can not be attributed to the natural and rapid breakdown of cAMP, as the phosphodiesterase inhibitor IBMX was present throughout the assay. Although these data seem controversial, other group has reported, in zebrafish Z3 cells [59], results which led to similar conclusions, indicating a complex system involving cAMP and clock genes.

## Discussion

### Melanopsin and peripheral clocks

For years the zebrafish has been used as a model in many areas of life sciences and in the last decade its use has expanded to the field of peripheral clocks. The discussion about peripheral clocks has gained even more importance by the fact that circadian system components may act in a reciprocal way, where peripheral and central clocks mutually interact to promote an adequate behavioral response. In mammals, for example, food availability exerts a powerful entrainment effect on behavior [60] and can reduce retinal input activation of cells that reside in the suprachiasmatic nucleus (SCN), promote transient disorganization of SCN outputs, and reduce sensitivity to SCN signals in hypothalamic sites responsible for integrating homeostatic and circadian information [61]. But in organisms such as *Drosophila melanogaster*, *Danio rerio* and *Xenopus laevis*, the impact of peripheral clocks can be even greater, since their bodies directly respond to light [33]–[36], [62], which makes the study of the mechanisms underlying peripheral clocks an intriguing issue.

Zebrafish have six *cry* genes, *cry1a*, *1b*, *2a*, *2b*, *3* and *4*, and four *per* genes, *per1a*, *1b*, *2* and *3*. All these genes exhibit cyclic expression pattern in light-dark cycle, which persists in constant dark, except *per2* [46]. However, only two are known to be light induced, *per2* and *cry1a* [49]. Both genes seem to have a critical role in resetting the circadian clock, whilst *per2* is also involved in the onset of the pineal circadian clock [55] and *cry1a* is a key element of the photoentrainment molecular machinery [56]. But how does the light signal reach these clock genes?

Tmts have been proposed to be the light sensing pigment that resets peripheral clocks in fish [25], [26]. In addition, there has been some speculation about one of the *cry* genes acting as a photoreceptor [36], [59], [63], and some data suggest that oxidative stress could be the signal that increases clock gene expression [64]; our results, however, point to the role of an opsin in the regulation of ZEM-2S clock genes.

Previous results from our laboratory have shown that the *Xenopus laevis* melanophore response to light occurs between 450 and 470 nm, and the opsin suggested to participate is one of the two melanopsins [20]. Five melanopsin genes have been described in *Danio rerio* [7], and quantitative PCR of ZEM-2S cells has demonstrated that all five are detectable in this cell line. One has to bear in mind, however, that cell lines may show altered gene expression, with native genes turned off, bringing caution to the interpretation of data obtained in cultured cells. Furthermore, ZEM-2S cells are derived from gastrula and in such an early developmental stage, some genes may have already been turned on, while others may still be off.

It is important to mention that in the blind cavefish, *Phreatichthys andruzzi*, the light-insensitive clock is entrainable by food. However, the ectopic expression of *opn4m-2* and *tmt* zebrafish homologues rescued the induction of a *Per2-Luc* reporter in the cavefish cells by blue (468 nm) or green (530 nm) light [26]. As it has been confirmed in *Danio rerio* that some melanopsin pigments are maximally sensitive to blue wavelengths [7], it is possible that melanopsin may be a good candidate to mediate entrainable light responses in zebrafish peripheral clocks. In this study, we have shown that light at wavelengths that fall within the absorbance spectra for melanopsin pigments that have been determined so far can modulate the expression of both *per1b* and *cry1b* and induce the expression of *per2* and *cry1a*. Nevertheless it has yet to be shown that melanopsin is directly responsible for these changes, and other pigments that are sensitive to blue light and share components of the signaling pathway being assayed may also be critical. Furthermore, recent work in zebrafish have shown that activation of the *per2* gene can be achieved by visible wavelengths other than blue, such as green (530 nm) and red (657 nm) [26], [65], and the participation of other opsins in the resetting of clock genes under white light cannot be discarded.

As previously stated, melanopsin shares both sequence identity and some similarities in signaling with invertebrate pigments compared to the other vertebrate opsin classes; for example, the involvement of a G_q/11_-type G protein and the phosphoinositide pathway. After the photostimulation of melanopsin, PLC is activated, and the ultimate event in the mammalian retinal ganglion cells is the influx of calcium through TRP channels, and action potentials [66]. Interestingly, inositol trisphosphate (IP_3_) is not necessary for the calcium rise. Our data demonstrates that light-evoked responses are blocked in the presence of the PLC inhibitor U-73122, thereby, confirming that PLC is fundamentally important in the photoactivation of clock genes in ZEM-2S cells. This inhibitor has a broad activity, with no specificity for any particular subtype of PLC. It has been accepted that PLCβ4 would be the isoform involved in melanopsin signaling due to its high expression in mammalian eye as compared to the other isoforms, by analogy with the invertebrate phototransduction, and because the knockout of PLCβ4 abolished the photoresponses in a subtype of ganglion cells in mice [66].

Besides PLC, various studies have shown that melanopsin activation leads to a transient rise in intracellular calcium [17]–[20], [67]. The concentration of cytoplasmic calcium is strongly regulated by the cell, and two classes of receptors mediate calcium release from the endoplasmic reticulum: inositol 1,4,5 trisphosphate (IP_3_) and rianodine receptors [68]. IP_3_, as well as diacylglycerol (DAG)), is a cleavage product of the membrane phospholipid phosphatidyl inositol-4,5-bisphosphate (PIP_2_), and its role in calcium release in response to light has been demonstrated in some studies [20], [24]. However, application of DAG and IP_3_ analogues did not induce photoresponses in melanopsin positive ganglion cells strongly suggesting that the ion channels may be regulated by the depletion of PIP_2_, or by polyunsaturated fatty acids released from DAG cleavage [66]. The inhibition of *per2* and *cry1a* expression by the chelator BAPTA-AM demonstrates the relevance of the calcium rise in the photoresponse of ZEM-2S cells.

Another important enzyme, PKC, has also been shown to play a role in the activation of clock genes in our model. It is well known that PKC comprises a large family of proteins, subdivided in three subfamilies: (i) classic PKC (cPKC), which are activated by calcium, DAG, phosphatidyl choline and phorbol esters; (ii) new PKC (nPKC), which are activated by the same compounds as cPKC, but calcium insensitive; and (iii) atypical PKC (aPKC), which are only activated by phosphatidyl serines [69]. The presence of a non-specific PKC inhibitor, Ro 31-8220, blocked the response to a blue light stimulus in ZEM-2S cells, and amongst all isoforms of PKC the most likely to participate in clock gene activation is PKCzeta [22]. In mice, the ablation of the PKCzeta gene (*Prkcz*) induces a similar phenotype to the melanopsin knockout mouse [22], and its presence has been reported in the zebrafish central nervous system [70].

Taken together, these two results (i.e. blue light-evoked responses and phosphoinositide pathway photoactivation) suggest that melanopsin may be a good candidate to induce *per2* and *cry1a* in ZEM-2S cells under these experimental conditions. Other possible candidates include va opsin and tmt opsin, but phylogenetic studies have shown that these two opsins exhibit higher sequence similarity to other vertebrate (non-melanopsin) opsin classes compared to invertebrate pigments [71], [72], suggesting they may signal via an activation of transducin (G_i/0_), phosphodiesterase and the hydrolysis of cyclic nucleotides. In fact, it has been demonstrated that the *opn3* family which includes *tmts* signal through G_i_/G_0_ proteins in the teleost *Takifugu rubripes* and the mosquito *Anopheles stephensi* [73]. There are many further opsin genes identified in the zebrafish genome (e.g. peropsin and *rgr*); however, at present very little is known about their expression patterns and putative physiological roles.

Cry proteins also sense blue light, but the data presented here do not agree with the known *cry* signaling. In *Drosophila melanogaster* the CRY protein undergoes a conformational change after light absorption by its flavin component, allowing activated CRY to interact with Timeless (TIM) and others factors to promote TIM proteolysis, resulting in the resetting of the circadian clock [74]. Therefore, CRY acts as a “light sensor” and directly resets the clock molecular machinery, without the activation of a signaling cascade. On the other hand, in zebrafish ZEM-2S cells, light can act through a signaling pathway (phosphoinositide) to modulate clock genes expression, making the resetting of the circadian clock by photosensitive Cry unlikely. However, the possibility of oxidative stress is still perfectly plausible. Pittendrigh [75] postulated a hypothesis called “escape-from-light”, where both light and temperature played major roles in the evolution of circadian organization, thus it would not be surprising that opsins (or other light detecting mechanisms) and oxidative stress co-exist in the same circadian system and even contribute to the same response (see discussion below).

### PLC pathway crosstalks

In excitable cells that express melanopsin, the ultimate event of the light-activated phosphoinositide pathway is usually the opening of the transient receptor potential channels subclass C (TRPC channels) and cell depolarization [76], but the potential link between the PLC signaling pathway and clock gene activation remains unknown.

In the SCN, cAMP seems to be an important second messenger triggering the expression of clock genes. The cyclic nucleotide activates PKA which is able, among other effects, to catalyze cAMP response element binding protein (CREB) phosphorylation in several models [56]. Cyclic AMP content fluctuates in the SCN [77], [78], and the *in vitro* application of its permeable analogue during the middle of the subjective day induces phase shifts [79]. More recently, cAMP signaling was proposed to be part of the molecular mechanism of the circadian clock itself and not just restricted to an involvement with the photoentrainment pathway [80]. Some of our data presented inconsistent or seemingly opposing results: namely that the quantification of cAMP suggested that this cyclic nucleotide is not required for the light-induced expression of clock genes, whereas the presence of an adenylyl cyclase inhibitor reduced *per2* and *cry1a* expression. Similar results were reported in a study with *Danio rerio* Z3 cells [59], in which the inhibition of PKA reduced the induction of *per2* expression by light. By contrast, forskolin, an adenylyl cyclase activator, did not induce *per2* expression. Given the literature reports and the data presented here, the role of cAMP in the modulation of clock genes remains unclear and needs to be further investigated.

On the other hand, several studies have suggested that clock gene activation might result from stimulation of the NO/cGMP cascade. Pharmacological and electrophysiological studies in the mammalian SCN demonstrated the importance of neuronal nitric oxide synthase (nNOS) in the circadian response to light as well as its association with CAMK II [81]–[88]. Similarly, our results indicate the participation of NOS and CAMK II in clock gene activation, since the presence of either inhibitor, L-NAME or KN-93, significantly reduced the photoinduced increase in expression of two representative clock genes.

An unexpected result was obtained with the guanylyl cyclase (GC) activator, YC-1. The classic NO pathway comprises the production of NO by NOS, followed by the stimulation of GC, an increase in cGMP levels and finally the activation of protein kinase G (PKG). However, the increase of cGMP promoted by YC-1 in ZEM-2S cells did not induce *per2* and *cry1a* expression. This is strong evidence that PKG activation is unrelated to the increase in expression for at least two clock genes, and that NO may act through a cGMP-independent pathway. Indeed, in neuronal cells, NO is capable of eliciting ERK phosphorylation without cGMP involvement [89], and may provide a pathway by which light, through NO production, could trigger the MAPK/ERK pathway [90].

Interestingly, the MAPK cascade is another pathway that appears to be involved with clock gene activation in ZEM-2S cells. MAPKs are activated by a variety of stimuli such as growth factors, cytokines, oncogenes and stressful conditions, and they are known to regulate cellular processes such as gene expression, differentiation and proliferation [91]. Moreover, MAPKs have been reported to play an important role in the formation of circadian rhythms in the SCN [92], [93]. Inhibition of ERK2 has been reported to block the circadian response to light [94]–[97], and the three family members, ERK1/2, p38 and c-Jun N-terminal kinase (JNK), exhibit diurnal and circadian changes in their activity in the SCN [98]. In zebrafish, the involvement of MAPKs and light in the induction of clock genes has been investigated [59], [65], [99]. Although not all in agreement, these studies all show the fundamental importance of the kinase in circadian entrainment: two reports show that MAPK has a positive effect under white light, since its inhibition decreases the induction of clock genes, but a latter study shows that, under blue light, MAPK acts negatively, where the presence of its inhibitor yielded a stronger and more sustained expression of *per2* and *cry1a*. Our data corroborate the first two reports, as an inhibitor of MEK (PD-98059) drastically reduced the enhancement of *per2* and *cry1a* expression by blue light.

These two signaling pathways, MAPK and NO, may act together, since both are involved with CREB phosphorylation in the SCN [90], [100]. In addition, NO is found to be necessary for ERK phosphorylating activity in primary cortical neuronal cultures [89]. The similarities between the signaling pathways in the mouse SCN and peripheral zebrafish cells suggest a preserved mechanism for the modulation of clock genes throughout vertebrate evolution. Such conserved mechanisms are obviously critical to cellular function and, therefore, illustrates an increasing need to better understand the resetting mechanisms that underpin clock gene expression in peripheral clocks.

### Oxidative stress and opsins

For many years scientists have been searching to identify the photoreceptor molecules that render zebrafish cells sensitive to light and to elucidate how they function to reset clock genes. There is supporting evidence for two possibilities, oxidative stress and opsins. Most data supporting oxidative stress suggest that flavin-containing oxidases are the light sensors responsible for circadian photoentrainment in zebrafish cells [64], [101]; however their functional role has yet to be demonstrated. Some studies have suggested that opsins such as Tmt may be the elusive photopigment [25], [26]; however, our results imply that Opn4 (or at least an opsin that shows some resemblance to the signaling pathway of melanopsin) may underpin photoentrainment in the zebrafish. Strengthening this possibility, light sensitivity of the cavefish *Phreatichthys andruzzii* clock is lost due to mutations in *tmt* and *opn4m-2*, and is rescued after transfection with zebrafish *tmt* and *opn4m-2* [26]. Since Tmt signaling has been proved to be via G_i_/G_0_ [73], our data on blue light signaling through phosphoinositide cascade point to Opn4.

Of course it is possible that these mechanisms, oxidative stress and opsin signaling, are not mutually exclusive of each other. Indeed, these two cellular processes seem to converge on the same signaling component, specifically MAPK in this case [59], [64], suggesting that these two mechanisms have probably evolved due to the same selective pressure, i.e. light associated to higher temperatures. The difference between these two signaling pathways lies on the molecules that activate the MAPK pathway: in oxidative stress activation relies on the action of reactive oxygen species (ROS) [64], [101], whereas the signaling pathway proposed here is based on the generation of reactive nitrogen species (RNS). A large number of reports indicate that RNS, as well as ROS, may act on signaling pathways through post-translational modifications, providing robust spatial and temporal control of protein conformation that result in the fine adjustment of a particular protein activity (for review, see [102], [103]). The physiological relevance of these two distinct systems may be justified by the levels of irradiance to which zebrafish are exposed.

At dusk and dawn, due to a lower level of irradiance for example, opsins may offer a better detection system for circadian entrainment, where just a single photon can trigger opsin activation [71], [72]. In this scenario, oxidative stress may be more effective under high irradiance levels or stressful light conditions, thus not only reinforcing circadian photoentrainment but linking it to photoreactive mechanisms such as DNA repair [101].
